# 6-Chloro-2*H*-1,4-benzoxazin-3(4*H*)-one

**DOI:** 10.1107/S1600536808035599

**Published:** 2008-11-20

**Authors:** Wen-Chang Zhuang, Yong-Sheng Xie

**Affiliations:** a School of Chemistry and Chemical Engineering, Xuzhou Institute of Technology, Xuzhou, Jiangsu 221006, People’s Republic of China; b School of Chemistry and Chemical Engineering, Shandong University, Jinan 250100, People’s Republic of China

## Abstract

In the title compound, C_8_H_6_ClNO_2_, the conformation of the six-membered heterocyclic ring is close to screw boat and the mol­ecules are linked *via* inter­molecular N—H⋯O hydrogen bonds along the *b* axis.

## Related literature

For biological activities of 1,4-benzoxazin-3(4*H*)-one derivatives, see: Huang *et al.* (2005 ▶); Macchiarulo *et al.* (2002 ▶). For a related structure, see: Pang *et al.* (2006 ▶).
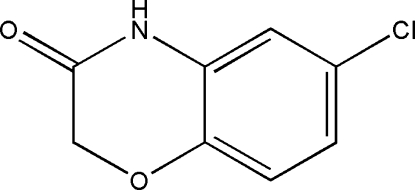


## Experimental

### 

#### Crystal data

C_8_H_6_ClNO_2_
*M* *_r_* = 183.59
Orthorhombic, 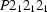

*a* = 4.5359 (6) Å
*b* = 7.700 (1) Å
*c* = 21.281 (3) Å
*V* = 743.28 (17) Å^3^
*Z* = 4
Mo *K*α radiation
μ = 0.46 mm^−1^
*T* = 273 (2) K
0.12 × 0.10 × 0.06 mm

#### Data collection

Bruker SMART CCD area-detector diffractometer
Absorption correction: multi-scan (*SADABS*; Bruker, 2002 ▶) *T*_min_ = 0.878, *T*_max_ = 0.973
3857 measured reflections
1314 independent reflections
1143 reflections with *I* > 2σ(*I*)
*R*_int_ = 0.047

#### Refinement

*R*[*F* ^2^ > 2σ(*F* ^2^)] = 0.038
*wR*(*F* ^2^) = 0.088
*S* = 1.07
1314 reflections
109 parameters
H-atom parameters constrained
Δρ_max_ = 0.16 e Å^−3^
Δρ_min_ = −0.21 e Å^−3^
Absolute structure: Flack (1983 ▶), 500 Friedel pairs
Flack parameter: 0.06 (11)

### 

Data collection: *SMART* (Bruker 2002 ▶); cell refinement: *SAINT* (Bruker 2002 ▶); data reduction: *SAINT*; program(s) used to solve structure: *SHELXS97* (Sheldrick, 2008 ▶); program(s) used to refine structure: *SHELXL97* (Sheldrick, 2008 ▶); molecular graphics: *XP* in *SHELXTL* (Sheldrick 2008 ▶); software used to prepare material for publication: *SHELXL97*.

## Supplementary Material

Crystal structure: contains datablocks I, global. DOI: 10.1107/S1600536808035599/wn2285sup1.cif

Structure factors: contains datablocks I. DOI: 10.1107/S1600536808035599/wn2285Isup2.hkl

Additional supplementary materials:  crystallographic information; 3D view; checkCIF report

## Figures and Tables

**Table 1:** Hydrogen-bond geometry (Å, °)

*D*—H⋯*A*	*D*—H	H⋯*A*	*D*⋯*A*	*D*—H⋯*A*
N1—H1⋯O1^i^	0.86	2.00	2.844 (3)	166

Symmetry code: (i) 
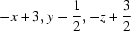
.

**Fig. 1.**
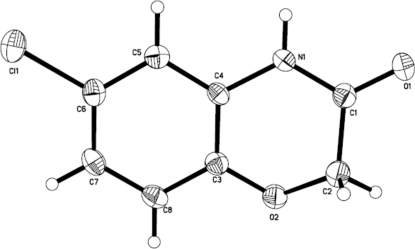
The molecular structure of the title compound, with displacement ellipsoids drawn at the 50% probability level. H atoms are shown as spheres of arbitrary radius.

## supplementary crystallographic information

### Comment

Benzo[1,4]oxazin-3(4*H*)-one derivatives are one of the important classes of heterocyclic compounds and have been shown to exhibit a wide range of biological activities such as herbicidal (Huang *et al.*, 2005) and antifungal (Macchiarulo *et al.*, 2002). We report here the crystal structure of 6-chloro-2*H*-benzo[*b*][1,4]oxazin-3(4*H*)-one.

The molecular structure is illustrated in Fig. 1. The conformation of the six-membered heterocyclic ring is close to screw boat, with atoms C1 and C2 out of the plane of the remaining four atoms by 0.301 (5) and 0.635 (5) Å, respectively. In a related compound containing the benzo[1,4]oxazin-3(4*H*)-one system (Pang *et al.*, 2006), the heterocyclic ring also adopts a screw boat conformation. The molecules are connected *via* N - H ··· O hydrogen bonding into chains along the *b* axis.

### Experimental

To a 25 ml round-bottomed flask equipped with a reflux condenser were added 2-chloro-*N*-(5-chloro-2-hydroxyphenyl)acetamide (2.19 g, 10 mmol), potassium carbonate (2.76 g, 20 mmol) and anhydrous DMF (20 ml). The resulting mixture was heated under reflux for 90 min. After this time, the reaction mixture was poured into 80 g of water, and stirred for 15 min. The mixture was extracted with ethyl acetate (2 *x* 20 ml). The ethyl acetate extract was washed with saturated brine (10 ml). After drying over Na_2_SO_4_, the solvent was removed under vacuum and a colourless solid was obtained in 80% yield (1.46 g). Suitable crystals were grown by evaporation of a CH_2_Cl_2 _solution at room temperature for 4 d.

### Refinement

All H atoms were positioned geometrically (N - H = 0.86 Å, aromatic C - H = 0.93 Å, methylene C - H = 0.97 Å) and refined using a riding model; *U*_iso_(H) = 1.2 *U*_eq_(C, N).

### Crystal data

**Table:**

C_8_H_6_ClNO_2_	*D*_x_ = 1.641 Mg m^−^^3^
*M**_r_* = 183.59	Mo *K*α radiation λ = 0.71073 Å
Orthorhombic, *P*2_1_2_1_2_1_	Cell parameters from 1287 reflections
*a* = 4.5359 (6) Å	θ = 3.3–24.4º
*b* = 7.700 (1) Å	µ = 0.46 mm^−^^1^
*c* = 21.281 (3) Å	*T* = 273 (2) K
*V* = 743.28 (17) Å^3^	Plate, colourless
*Z* = 4	0.12 × 0.10 × 0.06 mm
*F*_000_ = 376	

### Data collection

**Table:**

Bruker SMART CCD area-detector diffractometer	1314 independent reflections
Radiation source: fine-focus sealed tube	1143 reflections with *I* > 2σ(*I*)
Monochromator: graphite	*R*_int_ = 0.047
*T* = 273(2) K	θ_max_ = 25.0º
φ and ω scans	θ_min_ = 1.9º
Absorption correction: multi-scan(SADABS; Bruker, 2002)	*h* = −5→4
*T*_min_ = 0.878, *T*_max_ = 0.973	*k* = −8→9
3857 measured reflections	*l* = −25→23

### Refinement

**Table:**

Refinement on *F*^2^	Hydrogen site location: inferred from neighbouring sites
Least-squares matrix: full	H-atom parameters constrained
*R*[*F*^2^ > 2σ(*F*^2^)] = 0.038	*w* = 1/[σ^2^(*F*_o_^2^) + (0.0401*P*)^2^] where *P* = (*F*_o_^2^ + 2*F*_c_^2^)/3
*wR*(*F*^2^) = 0.088	(Δ/σ)_max_ < 0.001
*S* = 1.07	Δρ_max_ = 0.16 e Å^−^^3^
1314 reflections	Δρ_min_ = −0.20 e Å^−^^3^
109 parameters	Extinction correction: none
Primary atom site location: structure-invariant direct methods	Absolute structure: Flack (1983), 500 Friedel pairs
Secondary atom site location: difference Fourier map	Flack parameter: 0.06 (11)

### Special details

**Table:**

Geometry. All e.s.d.'s (except the e.s.d. in the dihedral angle between two l.s. planes) are estimated using the full covariance matrix. The cell e.s.d.'s are taken into account individually in the estimation of e.s.d.'s in distances, angles and torsion angles; correlations between e.s.d.'s in cell parameters are only used when they are defined by crystal symmetry. An approximate (isotropic) treatment of cell e.s.d.'s is used for estimating e.s.d.'s involving l.s. planes.
Refinement. Refinement of *F*^2^ against ALL reflections. The weighted *R*-factor *wR* and goodness of fit *S* are based on *F*^2^, conventional *R*-factors *R* are based on *F*, with *F* set to zero for negative *F*^2^. The threshold expression of *F*^2^ > σ(*F*^2^) is used only for calculating *R*-factors(gt) *etc*. and is not relevant to the choice of reflections for refinement. *R*-factors based on *F*^2^ are statistically about twice as large as those based on *F*, and *R*- factors based on ALL data will be even larger.

### Fractional atomic coordinates and isotropic or equivalent isotropic displacement parameters (Å^2^)

**Table:**

	*x*	*y*	*z*	*U*_iso_*/*U*_eq_	
Cl1	0.59509 (18)	0.16633 (10)	0.92672 (4)	0.0538 (3)	
O1	1.5486 (5)	0.7552 (2)	0.74891 (10)	0.0488 (6)	
O2	1.1177 (4)	0.8424 (2)	0.88354 (9)	0.0466 (6)	
N1	1.2830 (5)	0.5732 (3)	0.80882 (10)	0.0347 (6)	
H1	1.3651	0.4842	0.7918	0.042*	
C1	1.3640 (6)	0.7302 (3)	0.79030 (14)	0.0350 (7)	
C2	1.2111 (7)	0.8777 (3)	0.82142 (15)	0.0448 (8)	
H2A	1.3430	0.9768	0.8222	0.054*	
H2B	1.0404	0.9094	0.7965	0.054*	
C3	0.9870 (6)	0.6859 (3)	0.89160 (13)	0.0346 (7)	
C4	1.0683 (6)	0.5467 (3)	0.85521 (12)	0.0295 (6)	
C5	0.9496 (6)	0.3866 (3)	0.86535 (13)	0.0339 (6)	
H5	1.0043	0.2924	0.8406	0.041*	
C6	0.7466 (6)	0.3675 (3)	0.91299 (13)	0.0369 (7)	
C7	0.6661 (6)	0.5045 (4)	0.94990 (13)	0.0408 (7)	
H7	0.5317	0.4889	0.9824	0.049*	
C8	0.7839 (6)	0.6642 (4)	0.93876 (13)	0.0402 (7)	
H8	0.7267	0.7586	0.9631	0.048*	

### Atomic displacement parameters (Å^2^)

**Table:**

	*U*^11^	*U*^22^	*U*^33^	*U*^12^	*U*^13^	*U*^23^
Cl1	0.0588 (5)	0.0489 (4)	0.0537 (5)	−0.0086 (4)	0.0042 (4)	0.0129 (4)
O1	0.0582 (14)	0.0464 (11)	0.0420 (13)	−0.0049 (10)	0.0170 (12)	0.0038 (9)
O2	0.0600 (14)	0.0389 (10)	0.0410 (13)	−0.0064 (12)	0.0119 (11)	−0.0098 (9)
N1	0.0375 (13)	0.0348 (12)	0.0319 (14)	0.0014 (11)	0.0068 (11)	−0.0017 (10)
C1	0.0401 (17)	0.0378 (14)	0.0272 (16)	−0.0022 (13)	−0.0029 (14)	−0.0005 (12)
C2	0.0532 (19)	0.0390 (15)	0.0423 (19)	−0.0032 (14)	0.0061 (16)	0.0019 (14)
C3	0.0362 (16)	0.0372 (14)	0.0305 (16)	−0.0012 (13)	−0.0001 (12)	−0.0028 (12)
C4	0.0291 (14)	0.0372 (14)	0.0221 (14)	0.0016 (13)	0.0002 (13)	−0.0008 (11)
C5	0.0370 (15)	0.0365 (14)	0.0282 (15)	0.0021 (13)	−0.0049 (13)	−0.0006 (11)
C6	0.0357 (15)	0.0424 (15)	0.0327 (17)	−0.0011 (13)	−0.0026 (13)	0.0083 (13)
C7	0.0392 (18)	0.0534 (17)	0.0297 (17)	−0.0007 (15)	0.0057 (13)	0.0011 (15)
C8	0.0425 (16)	0.0464 (15)	0.0315 (17)	0.0058 (16)	0.0041 (13)	−0.0052 (14)

### Geometric parameters (Å, °)

**Table:**

Cl1—C6	1.720 (3)	C3—C8	1.373 (4)
O1—C1	1.231 (3)	C3—C4	1.373 (4)
O2—C3	1.354 (3)	C4—C5	1.362 (4)
O2—C2	1.414 (4)	C5—C6	1.377 (4)
N1—C1	1.324 (3)	C5—H5	0.9300
N1—C4	1.402 (3)	C6—C7	1.365 (4)
N1—H1	0.8600	C7—C8	1.362 (4)
C1—C2	1.486 (4)	C7—H7	0.9300
C2—H2A	0.9700	C8—H8	0.9300
C2—H2B	0.9700		
			
C3—O2—C2	114.9 (2)	C5—C4—C3	120.7 (3)
C1—N1—C4	122.4 (2)	C5—C4—N1	121.2 (2)
C1—N1—H1	118.8	C3—C4—N1	118.0 (2)
C4—N1—H1	118.8	C4—C5—C6	118.5 (3)
O1—C1—N1	123.0 (3)	C4—C5—H5	120.7
O1—C1—C2	121.1 (2)	C6—C5—H5	120.7
N1—C1—C2	115.8 (3)	C7—C6—C5	121.3 (3)
O2—C2—C1	114.2 (2)	C7—C6—Cl1	119.4 (2)
O2—C2—H2A	108.7	C5—C6—Cl1	119.2 (2)
C1—C2—H2A	108.7	C8—C7—C6	119.5 (3)
O2—C2—H2B	108.7	C8—C7—H7	120.2
C1—C2—H2B	108.7	C6—C7—H7	120.2
H2A—C2—H2B	107.6	C7—C8—C3	120.1 (3)
O2—C3—C8	119.7 (2)	C7—C8—H8	120.0
O2—C3—C4	120.4 (2)	C3—C8—H8	120.0
C8—C3—C4	119.8 (3)		
			
C4—N1—C1—O1	−178.8 (2)	C1—N1—C4—C5	167.7 (3)
C4—N1—C1—C2	−0.5 (4)	C1—N1—C4—C3	−14.2 (4)
C3—O2—C2—C1	−44.4 (3)	C3—C4—C5—C6	−0.1 (4)
O1—C1—C2—O2	−152.3 (3)	N1—C4—C5—C6	177.9 (2)
N1—C1—C2—O2	29.4 (4)	C4—C5—C6—C7	−0.5 (4)
C2—O2—C3—C8	−152.6 (3)	C4—C5—C6—Cl1	−179.8 (2)
C2—O2—C3—C4	31.1 (3)	C5—C6—C7—C8	1.3 (4)
O2—C3—C4—C5	176.3 (3)	Cl1—C6—C7—C8	−179.4 (2)
C8—C3—C4—C5	0.0 (4)	C6—C7—C8—C3	−1.4 (4)
O2—C3—C4—N1	−1.7 (4)	O2—C3—C8—C7	−175.6 (2)
C8—C3—C4—N1	−178.1 (2)	C4—C3—C8—C7	0.8 (4)

### Hydrogen-bond geometry (Å, °)

**Table:**

*D*—H···*A*	*D*—H	H···*A*	*D*···*A*	*D*—H···*A*
N1—H1···O1^i^	0.86	2.00	2.844 (3)	166

Symmetry codes: (i) −*x*+3, *y*−1/2, −*z*+3/2.
